# Detection, visualization and quantification of protein complexes in human Alzheimer’s disease brains using proximity ligation assay

**DOI:** 10.1038/s41598-023-38000-4

**Published:** 2023-07-24

**Authors:** Wilber Romero-Fernandez, Cristian Carvajal-Tapia, Alex Prusky, Ketaki A. Katdare, Emmeline Wang, Alena Shostak, Lissa Ventura-Antunes, Hannah J. Harmsen, Ethan S. Lippmann, Kjell Fuxe, Jason A. MacGurn, Dasiel O. Borroto-Escuela, Matthew S. Schrag

**Affiliations:** 1 Department of Neurology, Vanderbilt University Medical Center, MRBIII 465 21St Avenue S, Suite 6158, Nashville, TN 37240 USA; 2 Vanderbilt Brain Institute, Vanderbilt University, Nashville, TN 37232 USA; 3 Department of Pathology, Microbiology, and Immunology, Vanderbilt University Medical Center, Nashville, TN 37240 USA; 4 Department of Chemical and Biomolecular Engineering, Vanderbilt University, Nashville, TN 37235 USA; 5 Department of Neuroscience, Karolinska Institute, Solna, 17177 Stockholm, Sweden; 6 Department of Cell and Developmental Biology, Vanderbilt University, Nashville, TN 37240 USA; 7 Receptomics and Brain Disorders Lab, Department of Human Physiology, Sport and Exercise, Faculty of Medicine, University of Malaga, Edificio Lopez-Penalver, Jimenez Fraud 10, 29071 Málaga, Spain; 8 Vanderbilt Memory and Alzheimer’s Center, Vanderbilt University, Nashville, TN 37235 USA

**Keywords:** Ubiquitylated proteins, Ubiquitylation

## Abstract

Examination of healthy and diseased human brain is essential to translational neuroscience. Protein–protein interactions play a pivotal role in physiological and pathological processes, but their detection is difficult, especially in aged and fixed human brain tissue. We used the in-situ proximity ligation assay (PLA) to broaden the range of molecular interactions assessable in-situ in the human neuropathology. We adapted fluorescent in-situ PLA to detect ubiquitin-modified proteins in human brains with Alzheimer’s disease (AD), including approaches for the management of autofluorescence and quantification using a high-content image analysis system. We confirmed that phosphorylated microtubule-associated protein tau (Serine202, Threonine205) aggregates were modified by ubiquitin and that phospho-tau-ubiquitin complexes were increased in hippocampal and frontal cortex regions in AD compared to non-AD brains. Overall, we refined PLA for use in human neuropathology, which has revealed a profound change in the distribution of ubiquitin in AD brain and its association with characteristic tau pathologies.

## Introduction

Traditional histological techniques have been central to visualizing cellular connections and understanding the function of the brain. These approaches were fundamental to Dr. Alois Alzheimer’s discovery of his eponymous disease and formed the core of Santiago Ramón y Cajal’s Nobel prize winning work in the early part of the twentieth century. With the advent of immunohistochemical methods and progressively more refined microscopy platforms, the degree of molecular information that can be derived directly from intact brain is progressively expanding. However, interrogating protein–protein interactions (PPIs) and post-translational modifications (PTMs) of proteins in-situ in the brain remains difficult^1,2^. Consequently, many disease mechanisms are primarily studied in cellular and animal model systems and in homogenized samples. Nevertheless, confirming that potential disease mechanisms are relevant in their appropriate context in neuropathological studies remains critical to interrogating the molecular mechanisms of brain diseases in translational neuroscience. Moreover, PPIs are potential targets for therapeutic drugs, so visualization and quantification of PPIs may be a useful tool in drug discovery and development^3–6^.

In-situ proximity ligation assay (PLA) is a technique used to study PPIs. It was first described twenty years ago^7^ and was applied to detect PPIs in cultured cells in 2005–2006^8,9^. The approach produces a punctate fluorescent focus when two antibodies bind targets near each other. Each antibody in the pair is conjugated to complementary oligonucleotides. These oligomers can hybridize to form circular DNA that serves as a template for amplification which is hybridized with a brightly fluorescent-labeled oligonucleotide complementary to the amplified rolling circle product^10–13^. The resulting signal implies the two antibody probes are close enough that their targets likely form a protein complex. The maximal distance between the two antibodies that can generate a signal is estimated to be between 15 and 40 nm depending on the size of the oligomers and whether primary antibodies are directly conjugated to the oligomer, or if conjugated secondary antibodies are used. This range is comparable to (albeit slightly larger than) the proximity resolvable using Fluorescent Resonance Energy Transfer (FRET), but with superior sensitivity for low-abundance proteins^14^. Use of the fluorescent PLA format in human brain tissue has been limited due to the extent of autofluorescence and non-specific signals. In this technical report, we aimed to refine the PLA methodology for use in human brain to expand the range of PPIs which can be evaluated in-situ in human neuropathology. To do that, we visualized the ubiquitination of a phosphorylated form of tau protein.

Age-related changes in the brain, along with genetic, lifestyle and environmental factors can promote the accumulation of modified tau species in neurons. These changes are most dramatic in Alzheimer’s disease (AD) and related tauopathies^15–17^. Because neurofibrillary tangles (NFTs), large intraneuronal aggregates of hyperphosphorylated tau, strongly correlate with cognitive symptoms in AD, tau pathology can be considered a major toxic factor promoting neuronal network failure and neurodegeneration^18–20^.

Aggregated tau is known to undergo several PTMs^21^ including acetylation^22,23^, methylation^24,25^, SUMOylation^26^ and ubiquitination^23,24,27–29^. However, the significance of these modifications in diseased human brain tissue remains unclear due to the lack of methods which can allow their anatomic, cellular, and subcellular localization, detection, and quantification. For example, the role of polyubiquitination in the complex pathways associated with tau aggregation and propagation is incompletely understood but may contribute to axonal failure in AD. Developing advanced molecular imaging tools to visualize this process in the brain and characterize tau ubiquitination or other protein complexes may provide critical insights into the biology of AD brain. In this study, we describe a new approach that make the fluorescent PLA suitable for human neuropathology applications.

## Methods

### Human brain tissue

Brain tissue was obtained from the Vanderbilt Brain and Biospecimen Bank at Vanderbilt University Medical Center Nashville, Tennessee, USA (IRB# 180287). Written consent for brain donation was obtained from patients or their surrogate decision makers. Ethical approval was granted by Vanderbilt University Medical Center Institutional Review Board. The study has been carried out in accordance with The Code of Ethics of the World Medical Association (Declaration of Helsinki) for experiments involving human subjects. Demographics and neuropathological information for each tissue donor is shown in Supplementary Table 1.

### Brain tissue preparation

Human brain tissue was obtained at autopsy and immersion fixed in 10% formalin (ThermoFisher Scientific, Pittsburgh, PA) at 4 °C for 1–3 days. The fixative solution was then removed and the tissued rinsed with 1 × TBS (Corning, New York, NY) three times for 5 min each. The tissue was placed in sterile 10% sucrose (Sigma-Aldrich, St. Louis, MO)/1 × TBS/0.02% sodium azide (NaN_3_, Sigma-Aldrich) until tissue sank and then 30% sucrose/1 × TBS/0.02% NaN_3_ for overnight at 4 °C or until the tissue sank.

The tissue block was affixed to the stage of vibratome using cyanoacrylate cement and fully submerged in 1 × TBS. Hippocampal and frontal cortex sections were prepared at 50 µm thickness. Floating tissue sections were transferred to 15 mL Falcon tubes with antigen retrieval buffer (10 mM citric acid pH 6.0 (Sigma-Aldrich) containing 0.05% Tween-20 (Sigma-Aldrich)) and heated to 95 °C for 20 min in the block heater*.* Hippocampal and frontal lobe sections were then washed with 100 mM glycine (Sigma-Aldrich)/1 × TBS/0.1% Triton X-100 (Sigma-Aldrich) buffer for 30 min followed by permeabilization with 0.3% Triton X-100/1 × TBS buffer for 30 min and two washes for 5 min each with 1 × TBS at room temperature.

### Proximity ligation assay and immunohistochemistry

Protein–protein interaction assessment in postmortem human brain was performed using the Duolink® Proximity Ligation Assay (PLA) kit (Sigma-Aldrich), following the protocol as previously described^13,30^ with some modifications. Primary antibodies used were: Ubiquitin Recombinant Rabbit Monoclonal Antibody clone 10H4L21 (5 μg/mL, 701339; Invitrogen, Carlsbad, CA), and the Phospho-tau (Ser202, Thr205) Monoclonal Antibody (AT8) (5 μg/mL, MN1020; Invitrogen) that recognizes phosphorylated tau at serine 202 (Ser202) and threonine 205 (Thr205) and labels neurofibrillary tangles^31–33^. For alternative reagents and solutions see^13,30^.

Fixed free-floating sections were incubated with the provided blocking solution for 60 min at 37 °C, transferred to a 6-well plate in 1 × TBS/0.1% bovine serum albumin (Sigma-Aldrich) and placed under the LED lamp BESTVA DC Series 1200W LED Grow Light Full Spectrum at 4 °C overnight for photobleaching. The disappearance of autofluorescence was checked by fluorescent microscopy before continuing the assay. Sections were then incubated with the primary antibodies at 4 °C overnight and washed four times for 10 min each with buffer A (1 × TBS/0.05% Tween-20) under gentle agitation. Secondary antibodies/proximity probes were diluted with the same antibody diluent that was used for the primary antibodies and was applied to the sections for 90 min at 37 °C. Unbound proximity probes were removed by washing the samples four times for 10 min each with buffer A at room temperature under gentle agitation. Sections were incubated with ligation solution for 60 min at 37 °C, then washed twice for 10 min each with buffer A at room temperature. The rolling amplification-hybridization mixture was then added for 120 min at 37 °C.

Nuclei were stained with 0.1 µg/mL of 4′,6-diamidino-2-fenilindol (DAPI, Cayman Chemical, Ann Arbor, MI) for 15 min. Neuritic plaques, neurofibrillary tangles and related AD pathological structures were stained histologically using 1 µM Thiazine Red for 15 min (Chemsavers Inc, Bluefield, VA) followed by two washes for 10 min each at room temperature with serial dilutions of buffer B (300 mM NaCl/30 mM sodium citrate/high purity water), first pure buffer B, then 1:2, 1:10 and finally 1:100. The sections were placed on a microscope slide and mounted using an antifade mounting medium (VectaShield Vibrance®, Vector Laboratories Inc, Burlingame, CA).

Immunohistochemistry labeling was performed as previously described^34,35^. Shortly after incubation with the primary antibodies and extensive washing, the sections were incubated for 120 min with the following secondary antibodies: Donkey Anti-Rabbit IgG H&L Alexa Fluor® 594 (2 μg/mL Abcam, Waltham, MA) and Donkey Anti-Mouse IgG H&L Alexa Fluor® 488 (2 μg/mL, Abcam). AD pathological structures were stained for 15 min with 1 µM Thiazine Red (Chemsavers Inc.) or 5 µM 4,4′-[(2-methoxy-1,4-phenylene)di-(1*E*)-2,1-ethenediyl]bisphenol (MX-04) (Tocris, Minneapolis, MN) at room temperature. The sections were washed four times for 10 min each with buffer A and mounted on a microscope slide as described above.

### Confocal imaging and quantifications

The images were acquired using a Zeiss LSM 710 confocal laser-scanning microscope (Carl Zeiss AG, Germany). We used 20 × air/dry or 63 × oil objective and 10 μm z-stack scanning projections with a step interval of 1 μm, with a minimum resolution of 1200 × 1200 pixels.

For quantification, we compared two approaches, a traditional semi-manual quantification using *ImageJ* and an automated approach using the HCS Studio software associated with the Cell Insight CX7 high-content imaging system (ThermoFisher Scientific, Waltham, MA). For the semi-manual quantification, *ImageJ* was used to process each image into 8-bit greyscale images for each channel. The channel containing the PLA signal was thresholded until the PLA puncta were reliably isolated from the background forming a binary image. Overlapping PLA puncta were segmented using a watershed function and over/undersized puncta were excluded by defining the expected size range of PLA puncta. Size thresholding was adjusted for each cell line or experimental condition and estimated by measuring the diameter of isolated PLA puncta. The number of PLA puncta were then counted. Quantification of nuclei proceeds similarly; greyscale images were processed with a Gaussian blur, then thresholded as previously described with the additional of a “Fill Hole” function to optimize nuclear counting. Clumps of nuclei were again segmented, and the size range was defined by measuring the area of the smallest and largest cell in the field. The number of cells were then counted, and the summary result of PLA puncta/nuclei or PLA puncta/field was reported.

Analysis on the high-content imaging platform was performed by importing greyscale confocal images to the HCS Studio software (ThermoFisher Scientific). Images were analyzed using the General Spot Measurement Tool to quantify the number of PLA puncta per field. Thresholds for spot roundness, minimal and maximal spot intensity and size were comparable to those used in the semi-manual quantification.

### Human induced pluripotent stem cell (iPSC) maintenance and differentiation to neurons

CC3 iPSCs^36^ were maintained in E8 medium^37^on cell culture treated plates coated with growth factor reduced Matrigel (Corning). iPSCs were passaged at 60–80% confluence using Versene (Gibco, Billings, MT)^38^. iPSCs were differentiated into cortical glutamatergic neurons using previously described protocols^39^ with minor modifications^40^. iPSCs were washed with 1 × DPBS (Gibco) and incubated in Accutase (Gibco) for 3–5 min to obtain dissociated cells in suspension. Cells were collected by centrifugation, resuspended in E8 medium containing 10 µM Y27632 (Tocris) and plated onto growth factor reduced Matrigel coated plates at a density of 2.5 × 10^5^ cells/cm^2^. Approximately 24 h after seeding, the medium was changed to E6 medium^38^ containing 10 µM SB431542 (Tocris) and 0.4 µM LDN193189 (Tocris) for 5 days to induce neural differentiation^41^. Following induction, the media composition was gradually transitioned to N2 medium (DMEM/F12 medium (Gibco) supplemented with 1 × N2 supplement (Gibco) containing 10 µM SB431542 (Tocris) and 0.4 µM LDN193189 (Tocris)) for 5 more days. On the 11th day, cells were washed with 1 × DPBS (Gibco) and dissociated by incubating in Accutase (Gibco) for 30 min to 1 h. Cells were collected by centrifugation, resuspended in neuron medium containing 10 µM Y27632 (Tocris) and plated onto growth factor reduced Matrigel coated plates at a density of 1 × 10^5^ cells/cm^2^. Neuron medium was composed of a 1:1 mixture of N2 medium and B27 medium (Neurobasal medium (Gibco) containing 200 mM GlutaMAX (Gibco) and 1 × B27 supplement (Gibco) or 1 × SM1 neuronal supplement (Stem Cell Technologies, Vancouver, CA)). Media changes were performed approximately every 24 h for the next 20 days followed by media changes every 3–4 days thereafter. Neurons were used for experiments after at least 70 days of differentiation.iPSC neurons were plated in an 8-well chamber slide coated with 20 µg/mL poly-L-ornithine (Sigma-Aldrich), 20 µg/mL Laminin (Corning) and 10 µg/cm^2^ collagen IV (Sigma-Aldrich) before performing the PLA as described above. After PLA, the neurons were counterstained with Alexa Fluor® 488 Anti-beta III Tubulin antibody, clone 2G10 (5 μg/mL, Abcam).

### Statistical analyses

The number of human brain donors (*n*) represented in each group is indicated in the figure legends. All data were analyzed using GraphPad Prism 9.0 (GraphPad Software Inc., San Diego, CA). An unpaired *t*-test was used to evaluate the two groups. The *p* value < 0.05 and lower was considered significant.

### Ethics approval and consent to participate

Brain tissue was obtained from the Vanderbilt Brain and Biospecimen Bank at Vanderbilt University Medical Center Nashville, Tennessee, USA (IRB# 180287). Informed consent for brain donation was obtained from patients or their surrogate decision makers. Ethical approval was granted by Vanderbilt University Medical Center Institutional Review Board. The study has been carried out in accordance with The Code of Ethics of the World Medical Association (Declaration of Helsinki) for experiments involving human subjects.

## Results

### Aggregated phosphorylated tau and ubiquitin distribution in AD hippocampus

**Figure 1:**
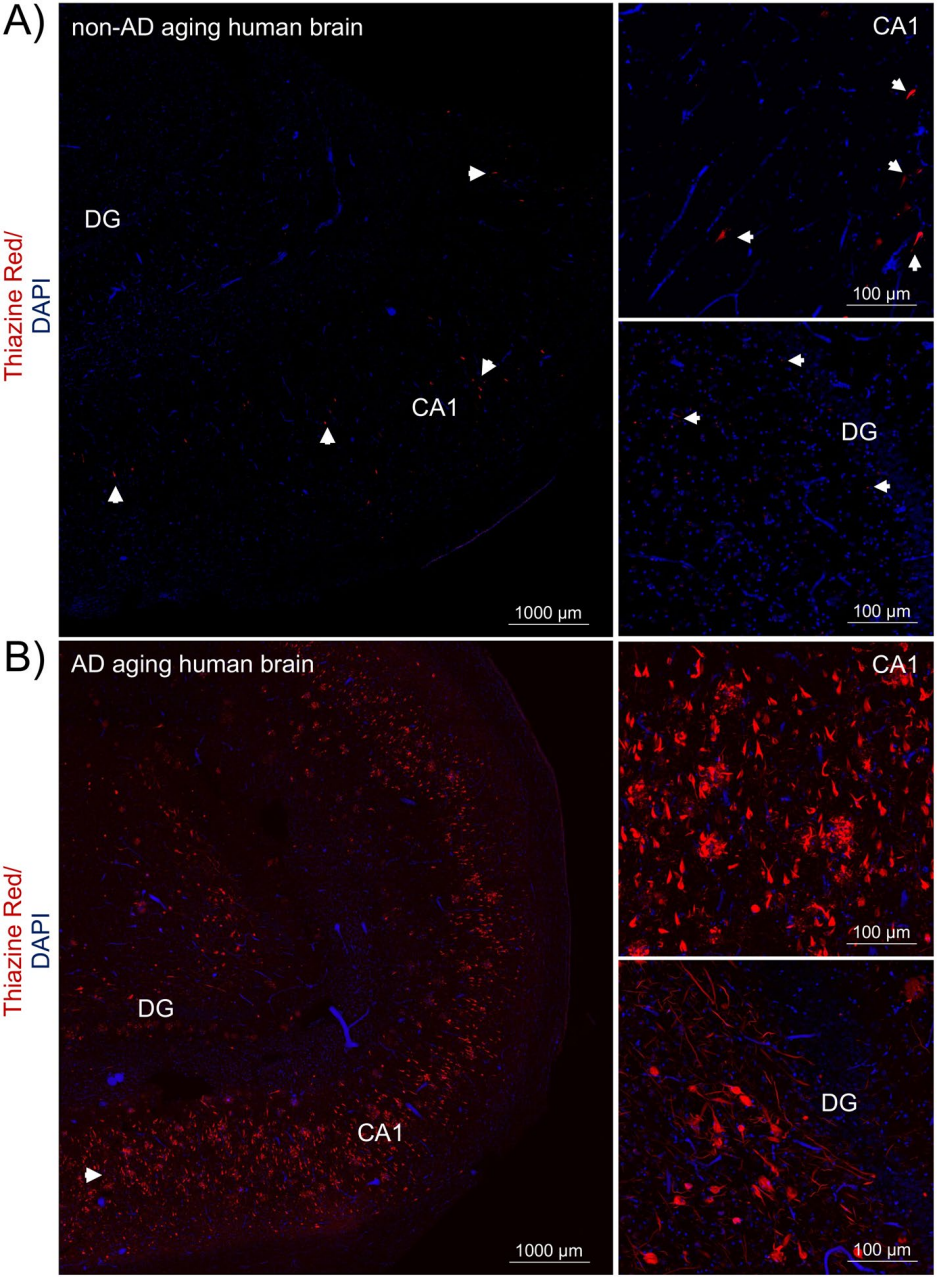
Large tau inclusions distinguish aging AD from aging human brains. This figure shows the early stage tauopathy seen in areas of human hippocampus in a “normal aging” (non-AD) brain without cognitive impairment compared to the advanced pathology seen in symptomatic AD. (**A**) Lower and higher magnification images of non-AD brain sections, *n* = 4, and (**B**) AD brain sections, *n* = 4. The two-channel merged representative images were produced from 10 μm z-stack scanning projections with a step interval of 1 μm. Nuclei were stained with DAPI (*blue*) and β-amyloid and neuritic plaques, neurofibrillary tangles and other tau aggregates were stained with Thiazine Red (*red, see arrows*). The scale bars are indicated. *Abbreviations*: **C**A1, cornu ammonis 1; DG, dentate gyrus.

We performed a histopathological assessment to test the specificity of antibodies in fixed free-floating hippocampal and frontal cortex sections, respectively. Fluorescent staining of hallmark AD pathological structures in hippocampus tissue is shown in Fig. 1 and Supplementary Fig. 1. For most of the non-AD aging brain sections analyzed, tau aggregates were essentially absent, although a few cognitively intact brain sections occasionally had neurofibrillary tangles (NFTs) (Fig. 1A and Supplementary Fig. 1A). In AD aging brains, abundant NFTs in neuronal cell bodies, neuropil threads and dystrophic neurites around neuritic plaques were observed, as is well known (Fig. 1B and Supplementary Fig. 1B).

**Figure 2:**
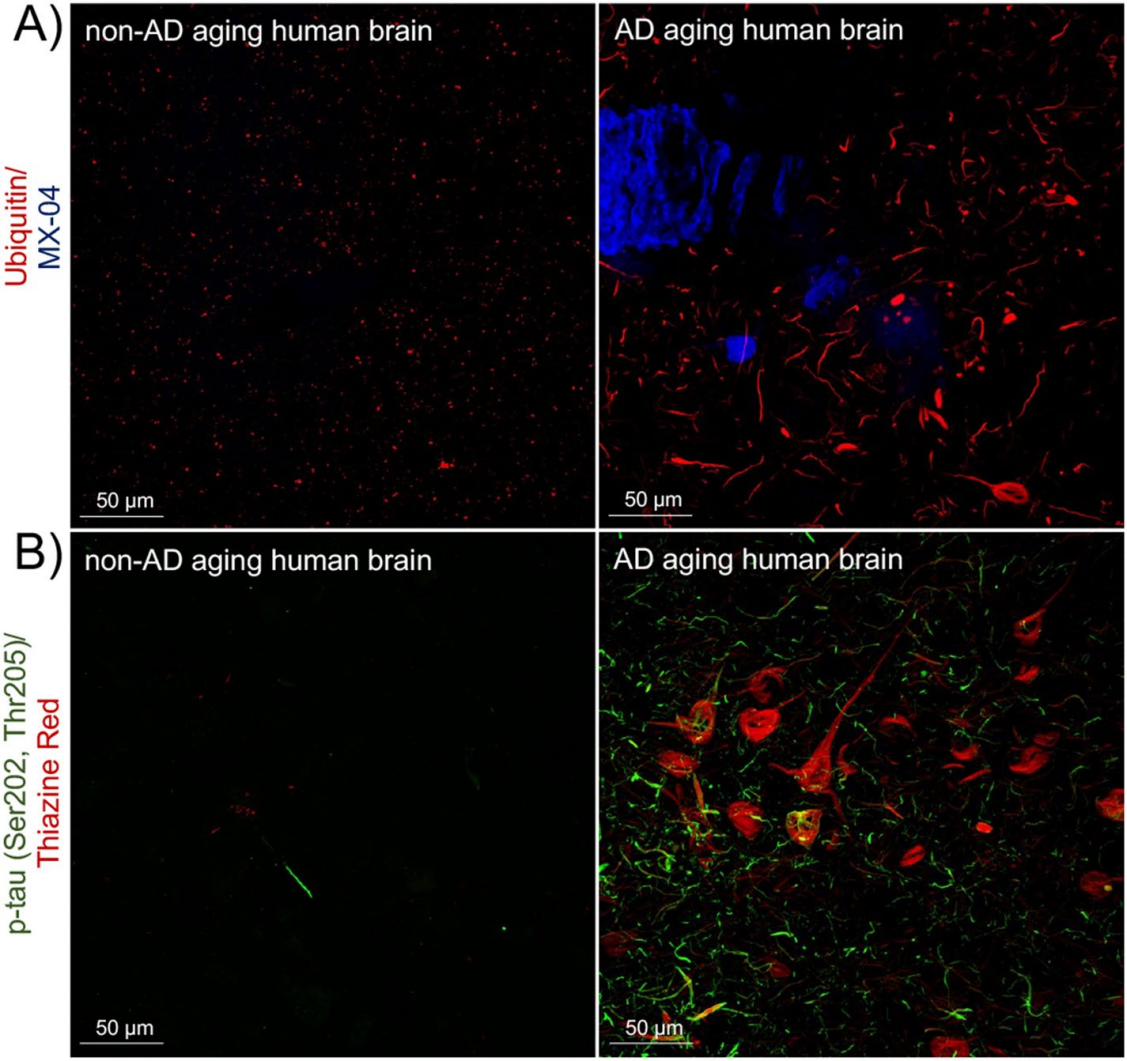
Specific immunolabeling revealed ubiquitin and phosphorylated tau in frontal cortex sections. (**A**) Immunolabeling of ubiquitin (*red*) in non-AD brain (*left panel*) and AD (*right panel*), *n* = 4, is shown. Neuritic plaques, neurofibrillary tangles and other tau aggregates were stained with MX-04 (*blue*). (**B**) Immunolabeling of p-tau (Ser202, Thr205) (*green*) in non-AD brain (*left panel*) and AD (*right panel*), *n* = 4, is shown. Neuritic plaques, neurofibrillary tangles and other tau aggregates were stained with Thiazine Red (*red*). The two-channel merged representative images were produced from 10 μm z-stack scanning projections with a step interval of 1 μm. The scale bars are indicated.

We evaluated the distribution of ubiquitin and p-tau in “normal” non-AD and in AD human brains (Fig. 2). In healthy brains and those with non-tauopathy disorders (like cerebral amyloid angiopathy), ubiquitin was detected primarily in puncta, mostly in a perinuclear distribution, likely representing physiological proteostatic mechanisms (Fig. 2A, *left panel*). The degree of change in the abundance and distribution of ubiquitin in AD brain is striking; ubiquitin-stained twisted structures in occasional neuronal cell bodies in a pattern typical of neurofibrillary tangles and linear areas resemble neuropil threads (Fig. 2A, *right panel*). Phosphorylated-tau immunohistochemistry in non-AD and AD brains was comparable to previous reports^42^ and is shown in Fig. 2B. The overall level of immunoreactivity in AD samples was much higher (Fig. 2B, *right panel*). In view of a similar distribution between ubiquitin and phosphorylated tau, we next sought to confirm the existence of p-tau (Ser202, Thr205)-ubiquitin complexes by PLA.

### Assessment of direct interaction between ubiquitin and phosphorylated tau in-situ

**Figure 3:**
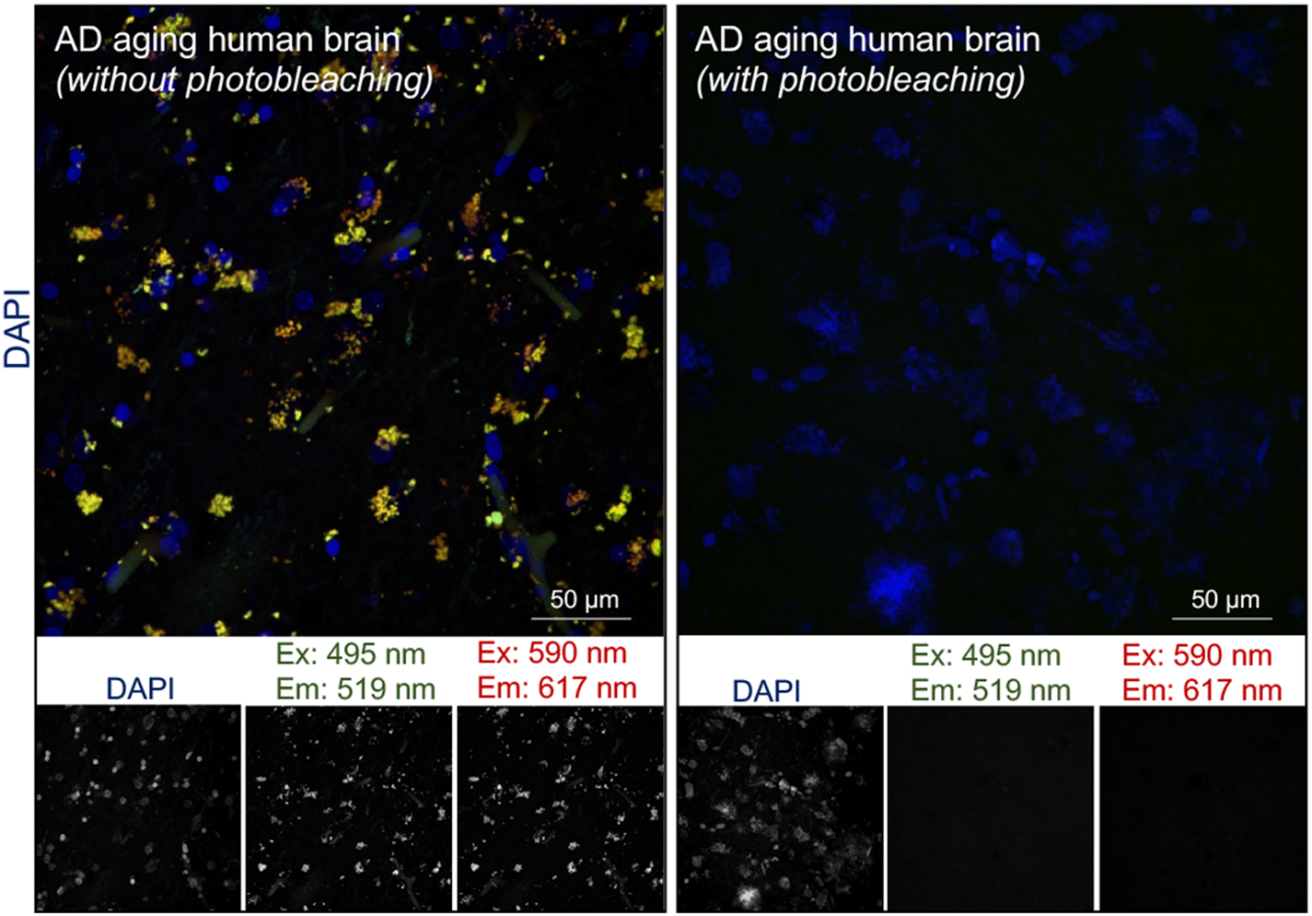
Optimized photobleaching abolishes intrinsic autofluorescence in human brain tissue. Autofluorescence in the emission spectrum of two commonly used fluorophores are shown in human brain sections without- and with-photobleaching. Dense fluorescent aggregates observed in the soma in sections without photobleaching (*right panel*) were abolished with photobleaching (*left panel*). Split channels of fluorescent excitation (*Ex*)/emission (*Em*) spectrum corresponding to green and red wavelengths are shown. Nuclei were stained with DAPI (*blue*), *n* = 3. The scale bars are indicated.

To date, in-situ PLA reports from the postmortem human brain remain scarce, in part due to technical difficulties such as intrinsic tissue autofluorescence which is exacerbated during fixation. We found that photobleaching using a broad-spectrum LED array can virtually abolish the autofluorescence (Fig. 3). The background observed with either 488 or 594-nm excitation (Fig. 3, *left panel*) was removed without tissue damage (Fig. 3B, *right panel*). Establishing a quiescent background is essential to obtaining specific PLA labeling with adequate signal-to-noise ratio for precise quantification (Fig. 3B, *right panel*).

**Figure 4:**
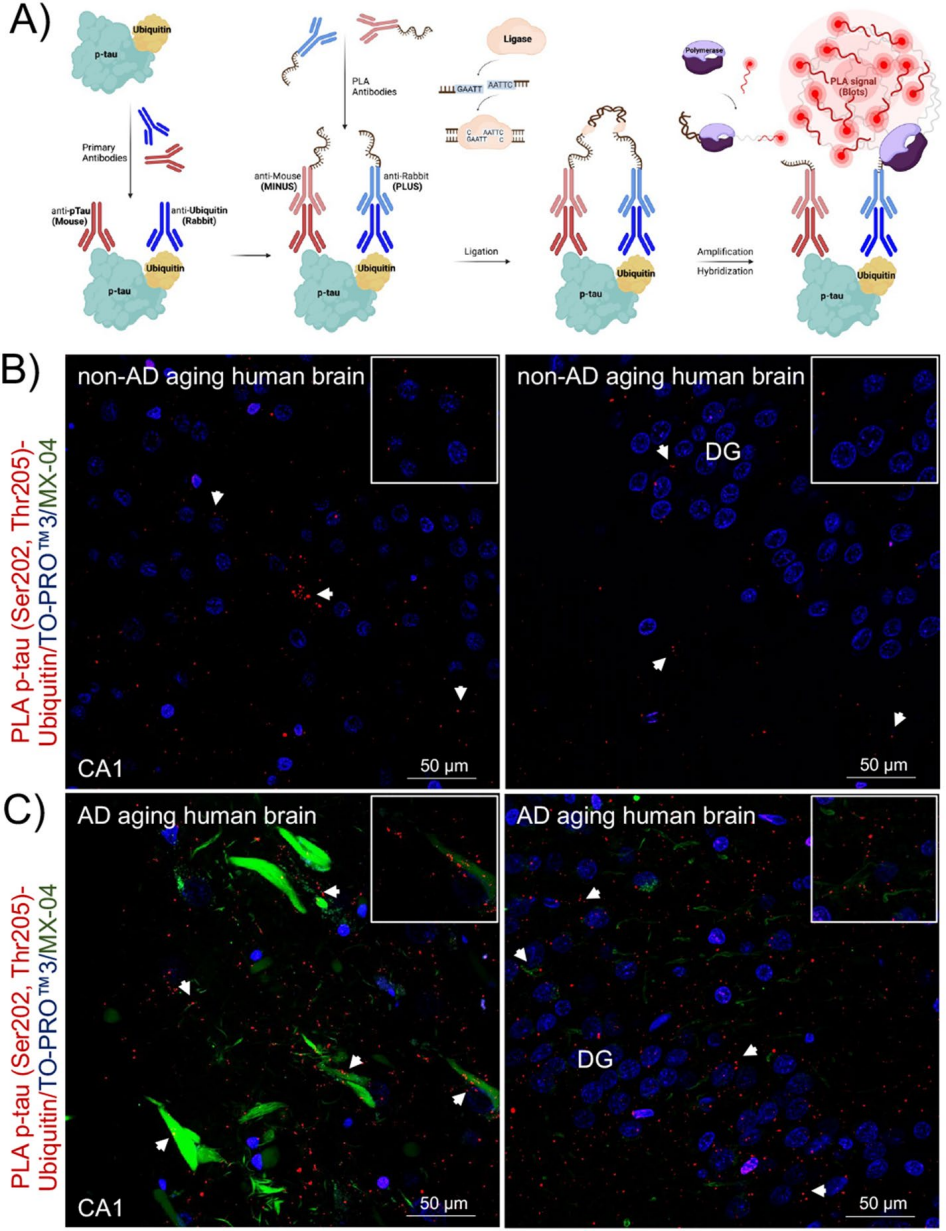
*In-situ* PLA detected protein complexes between p-tau and ubiquitin in hippocampal sections from non-AD and AD human brains. (**A**) Schematic representation of the PLA. First, a pair of well-validated primary antibodies binds target proteins. Second, a pair of secondary species-specific antibodies conjugated to complementary oligonucleotides (ssDNA, MINUS and PLUS) recognize the primary antibodies. If targets are in proximity (≤ 40 nm), a circular ssDNA will form. A ligase facilitates the hybridization of oligonucleotides to form a double strand acting as a template for rolling circle amplification. Finally, a polymerase generates an amplified rolling circle product that is hybridized by fluorescently labeled probes to generate a specific PLA signal *(red puncta)*. (**B**) Proximity ligation puncta indicated by arrows represent the protein complexes between p-tau (Ser202, Thr205) and ubiquitin found in areas of hippocampus in (**B**) non-AD, *n* = 3, and (C) AD, *n* = 3, sections. The three-channel merged representative images were produced from 10 μm z-stack scanning projections with a step interval of 1 μm. Nuclei were stained with TO-PRO^TM3^ (*blue*), and tau pathology and β-amyloid aggregates were stained with MX-04 (*green*). The scale bars are indicated. *Abbreviations*: CA1, cornu ammonis 1; DG, dentate gyrus.

Since the hippocampal formation is vulnerable to NFTs we first focused on evaluating the presence of p-tau-ubiquitin complexes in this region. The approach to labeling the target interaction is outlined in Figure 4A. Neurological control tissue from fixed human post-mortem specimens had well-defined PLA signals with minimal background staining primarily restricted to the nucleus (Fig. 4). In non-AD brains, the PLA signal corresponding to p-tau-ubiquitin complexes was sparse in areas of hippocampus (Fig. 4B). A clear increase of PLA signal was observed in the same areas in AD brains (Fig. 4C).

**Figure 5:**
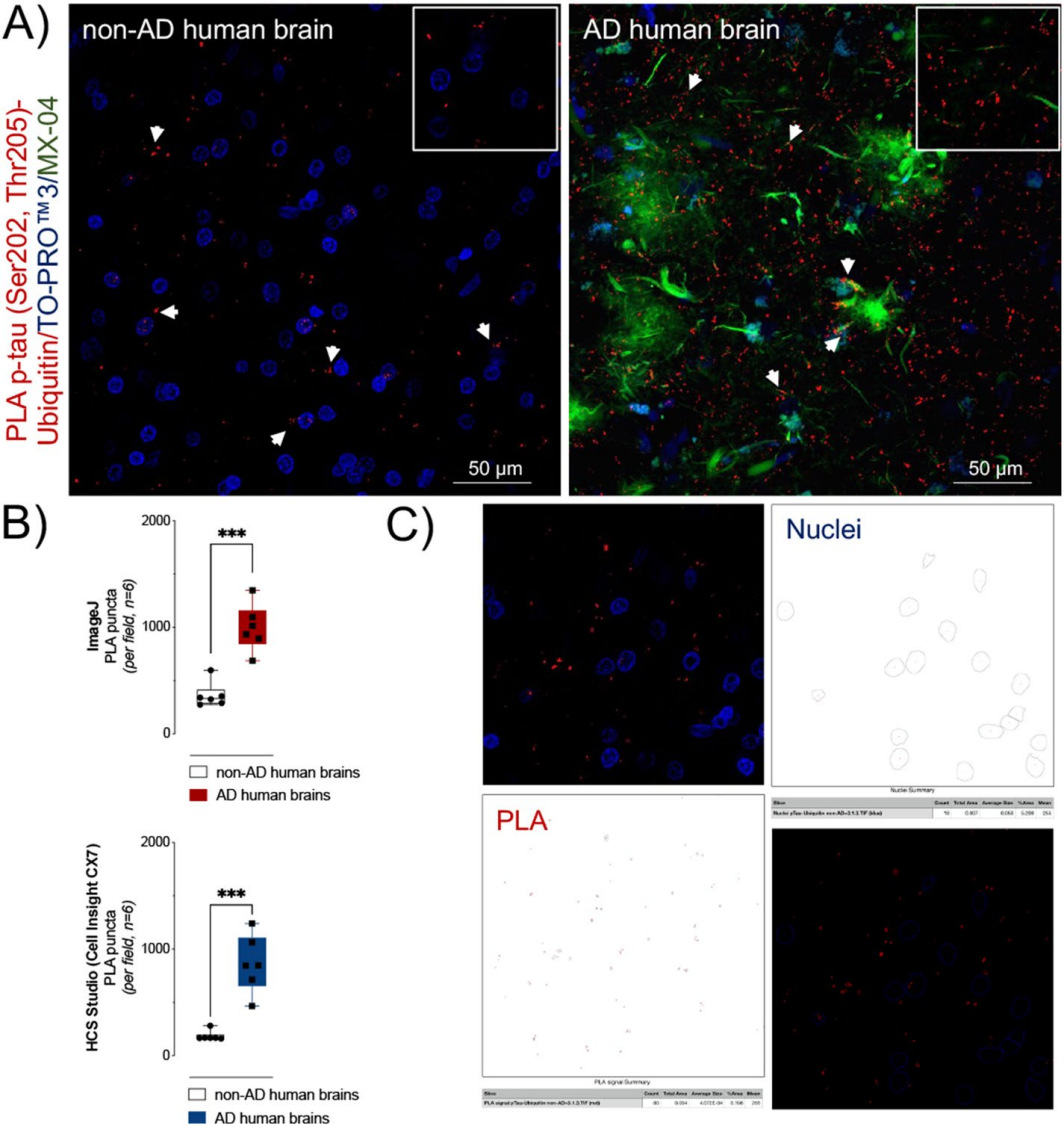
p-tau (Ser202, Thr205) modified with ubiquitin was significantly higher in AD frontal lobe tissue compared to non-AD. Quantification of p-tau (Ser202, Thr205) modified with ubiquitin in frontal cortex sections from non-AD and AD human brains. (**A**) Proximity ligation puncta (*red clusters*) indicated by arrows represent the protein complexes between p-tau (Ser202, Thr205) and ubiquitin found in non-AD (*left panel*) and AD (*right panel*), *n* = 6. Nuclei were stained with TO-PRO^TM^3 (*blue*) and tau and β-amyloid aggregates with MX-04 (*green*). The scale bars are indicated. (**B**) Upper panel (*ImageJ*): data presented (*PLA signal per field*) are means ± S.E.M., *n* = 6 individual brains, each with five images comprising a 10 μm z-stack of 224.92 µm × 224.92 µm scanning projections with a step interval of 1 μm. ***p < 0.001 by Unpaired *t*-test*.* Lower Panel: an alternative approach to quantification using the HCS Studio software produces a comparable result. ***p < 0.001 by Unpaired *t*-test*.* (**C**) Summary schematic representation of PLA data analysis using *ImageJ* (*for details see methods*). First, confocal microscopy images (*upper left panel*) were split to maximum intensity projection images of nuclei (DAPI) and protein complexes (PLA puncta). For each fluorescent channel, we performed conversion to grayscale, thresholding to create a binary output and finally watershed transformation to resolve individual particles for analysis of the DAPI (*upper right panel*) and PLA puncta (*lower left panel*). Composite images containing nuclear staining and PLA puncta enable the calculation of the number of PLA puncta per nuclei (*lower right panel*).

We quantified the relative number of fluorescent foci, which we term “PLA puncta”, in the frontal cortex (Fig. 5). Similar to the hippocampus, PLA puncta corresponding to p-tau-ubiquitin complexes was sparse in the frontal lobe of non-AD brains (Fig. 5A, *left panel*). Significantly greater levels of PLA puncta were observed in AD brains relative to non-AD brains (Fig. 5A, *right panel*: 996 ± 90 PLA puncta/field in AD vs. 363 ± 48 PLA puncta/field in neurological controls, *n* = 6, *p* < 0.0001) as determined by the quantification of the amount of PLA puncta per field using *ImageJ* (Fig. 5B *upper panel*). Automated quantification using the HCS Studio software associated with the Cell Insight CX7 high-content imaging system showed a comparable increase in the PLA signal, 862 ± 110 PLA puncta/field vs. 185 ± 19 PLA puncta/field, *n* = 6 (Fig. 5B *lower panel*). The approach to quantify PLA puncta using *ImageJ* is outlined in Fig. 5C and using the HCS Studio software in Supplementary Fig. 2.

**Figure 6:**
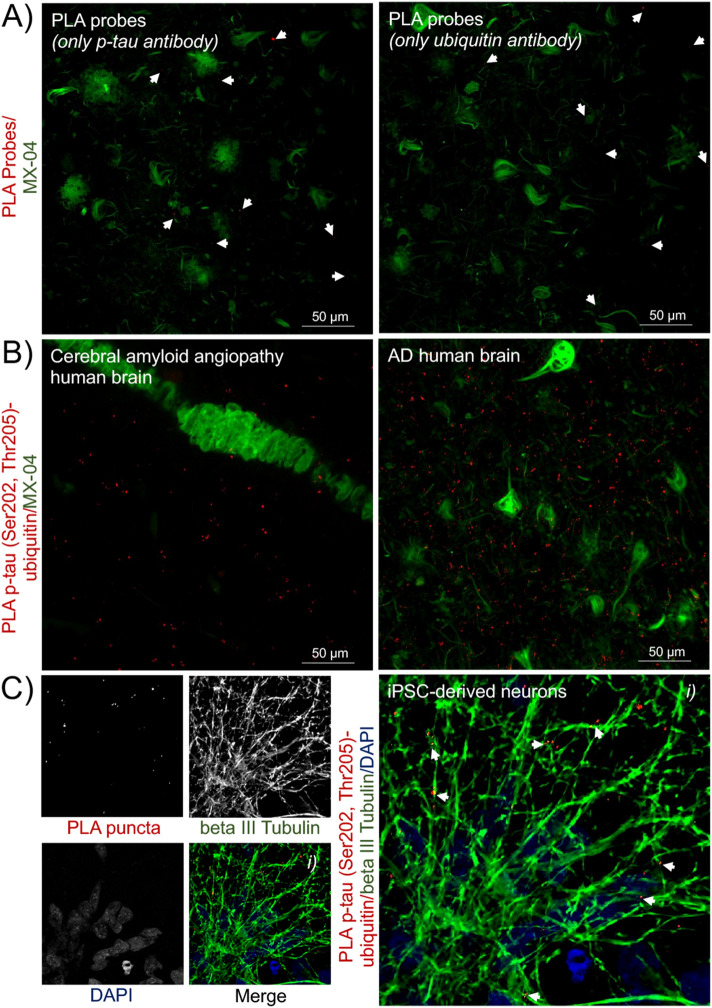
Technical and biological controls validated the *in-situ* PLA technique. (**A**) As technical controls for the PLA probes and procedure, PLA puncta (*red*) were evaluated while excluding one of the primary antibodies to assess non-specific background labelling. Representative images show the background PLA signals when using only the p-tau (Ser202, Thr205) antibody (*left panel*) or ubiquitin antibody (*right panel*). (**B**) As a biological control, we evaluated PLA puncta (*red*) detected in brain sections with cerebral amyloid angiopathy, but without or with low levels of tauopathy, *n* = 3. PLA signals were not associated with β-amyloid deposits (*left panel*) and less abundant than in tissue with frequent tau inclusions (*right panel*). Tau pathology and β-amyloid aggregates were stained with MX-04 (*green*). (**C**) As a final control, we show that the PLA procedure used in tissue sections remains functional in cultured iPSC-derived neurons, here stained via immunocytochemistry with beta III tubulin (*green*) and PLA showing p-tau (Ser202, Thr205)-ubiquitin complexes (*red*), *n* = 3. Nuclei were stained with DAPI (*blue*). The two- or three-channel merged representative images were produced from 10 μm z-stack scanning projections with a step interval of 1 μm. The scale bars are indicated.

Technical controls (Fig. 6A) using only the primary p-tau (Fig. 6A, *left panel*) or ubiquitin (Fig. 6A, *right panel*) antibodies displayed few and weak background PLA signals. For biological controls (Fig. 6B), we used brain sections from donors with cerebral amyloid angiopathy (CAA) without tauopathy or with low levels of tau pathology (*n* = 3). Sections with CAA showed PLA signals similar to those obtained in other non-AD samples (Fig. 6B, *left panel*) and much lower PLA signals than sections with severe tauopathy (Fig. 6B, *right panel*). The PLA signal in CAA did not associate with β-amyloid deposition (Fig. 6B, *left panel*). Because PLA has been validated in cell models, the detection of p-tau (Ser202, Thr205)-ubiquitin complexes in iPSC-derived neurons (using a cell line from a neurologically normal donor) was used as a positive control. PLA for the interaction of p-tau (Ser202, Thr205) with ubiquitin are primarily in neuronal processes as evidenced by their co-localization with beta III tubulin (Fig. 6C).

## Discussion

This technical report presents an adapted fluorescent in-situ PLA assay for use in human neuropathology which we applied to study ubiquitination of tau in postmortem human brains with AD. We were able to visualize phosphorylated tau (Serine202, Threonine205) aggregates modified by ubiquitin in brain tissue. Phospho-tau-ubiquitin complexes were increased in hippocampal and frontal cortex regions in AD compared to non-AD brains and were closely associated with tangles and neuropil threads. While ubiquitination of phosphorylated tau has been known for many years, these novel images of phospho-tau-ubiquitin complexes in the AD brain demonstrate the full extent of this neuropathology.

Since PLA puncta are produced as the result of binding sites which are separated by no more than 10 to 40 nm (depending on the details of the approach), it is reasonable to infer that this signal may originate from the same tau molecule, although we cannot completely rule out that a fraction of the PLA puncta come from coincidental proximity.

Prior uses of the fluorescent PLA in human neuropathological specimens are scarce^43^ and limited to brightfield preparations which have decreased sensitivity^44,45^, but are not impacted by tissue autofluorescence. Our optimized protocol includes a photoclearance step which abolishes the background autofluorescence from lipofuscin and other pigments that accumulate in the soma. Alternative approaches to controlling autofluorescence, like treatment with solutions containing copper containing solutions or dark pigments^46,47^, lead to tissue and/or antigen damage or mask specific PLA puncta which may be undesirable when attempting to detect a truthful number of protein complexes or sensitively quantitate low-abundance protein modifications or PPIs.

In addition to pristine control of autofluorescence, proximity ligation assays procedures require selective/specific and well validated antibodies. PLA has been criticized because the results use proximity as a surrogate for detecting PPIs^48^. It is possible that proteins may be incidentally in proximity without directly interacting, but this is primarily a concern under non-physiological conditions, such as when proteins are artificially overexpressed. It is likely that under the conditions present when evaluating brain tissue, the limitations of the assay are less problematic than in highly manipulated model systems. Nevertheless, the use of appropriate controls is essential for the accurate interpretation of the results, including defining the distribution and abundance of the individual proteins being studied. PLA does not replace biochemical studies proving the presence of a molecular interactions or modifications, but it is particularly valuable for visualizing molecular interactions or post-translational modifications in situ in tissue. Here, we used primary p-tau or ubiquitin antibodies alone as technical controls and brain sections from donors with CAA (without tauopathy or with low levels of tau pathology) as a biological control and both displayed few and weak background PLA signals.

The bright punctate signals resulting from in-situ PLA analysis are amenable to a range of quantitative approaches. Here, we found the technique was suitable for analysis in a high-content imaging system with results from human tissue. The coupling of PLA to high-content analysis greatly increases the rigor and efficiency of this method. In applying this technique, we found that phosphorylated tau associates with ubiquitin and accumulates in neurons in AD. This approach can be combined with histological or immunohistochemical stains to define the cell type, subcellular localization and other contextual features associated with a PPIs. Additionally, the compatibility of in-situ human brain PLA with high content imaging platforms may enable its use in medium and high-throughput applications. This report shows the fluorescent PLA technique can be a valuable tool to identify ubiquitin-substrate proteins, and while we focused on tau, the assay can be easily modified according to the PPIs or PTMs of interest.

Understanding the dysregulation of proteostasis in neurodegenerative disorders is an emerging research priority, as most neurodegenerative disorders are marked by the accumulation of protein aggregates of one form or another. Profound dysregulation of lysosomal function has been observed in Alzheimer’s disease and ubiquitin–proteasome system (UPS) deficiencies have been also reported in AD, Parkinson’s disease, frontotemporal dementia, and amyotrophic lateral sclerosis^49,50^. In fact, as we show here and others have reported, the pathological accumulation of ubiquitin is remarkably abundant in AD^29^. Polyubiquitination of proteins is best known for its role in facilitating protein degradation by the UPS and is accomplished by covalent crosslinking of a lysine residues in a chain of ubiquitin moieties to a lysine residue on a target protein. Variation in the linkage type of polyubiquitin chains can alter the fate of the tagged protein^51^. There are several different linkage sites on ubiquitin—the best-known being K48-polyubiquitination which targets proteins for degradation by the UPS pathway^52,53^ and K6, K27, K33 which are related to immunity, cell proliferation, and DNA damage repair^54^. Further, K63-polyubiquitination directs proteins to the autophagy lysosomal pathway (ALP) but does not necessarily result in protein degradation.

The precise mechanism(s) at play here at not yet known and could be clarified in future work by defining the specific ubiquitin linkages in phosphorylated tau aggregates. A form of aggregated tau in neurofibrillary tangles in AD, Paired Helical Filaments (PHF)-tau, is suggested to be polyubiquitinated via not only K48 linkages, but also via non-canonical K6- and K11- linkages^28^, whereas soluble tau can be ubiquitinated by K63^27^. The method presented in this report could be used to specifically detect which lysine residues are modified, providing information regarding whether the modification will result in degradative or non-degradative outcomes. Despite the rapid metabolism of ubiquitin-tagged proteins in healthy neurons^55^, our assay was able to detect ubiquitin conjugates in native conditions as evidenced by the PLA signals obtained in non-AD tissue. This likely indicates that under physiological conditions, the UPS controls tau synthesis, folding, and/or trafficking as well as degradation. The significant increase in PLA signal observed in AD brains demonstrates a defect in tau-related proteostasis in AD and is worthy of further study.

## Conclusions

We conclude that ex-vivo PLA provides valuable information about protein complexes and PTMs while preserving spatial information and has some advantages compared biochemical and biophysical methods previously reported. This approach does not require transient protein overexpression, insertion of tags to facilitate immunoprecipitation or UPS inhibition to prevent the rapid metabolism of the ubiquitin-tagged proteins. The combination of PLA and high-content image analysis makes the approach rigorously quantitative and efficient. Incorporating this advanced molecular imaging technique into the repertoire of neuropathological tools will broaden the range of molecular information that can be derived from human brain tissue.

## Supplementary Information

Supplementary Information.

## Data Availability

The datasets used in the current study are available from the corresponding author on reasonable request.

## Abbreviations

PLA: Proximity ligation assay
AD: Alzheimer’s disease
PPIs: Protein–protein interactions
PTMs: Post-translational modifications
NFTs: Neurofibrillary tangles
iPSC: Human induced pluripotent stem cell
UPS: Ubiquitin–proteasome system
CA1: Cornu ammonis 1
DG: Dentate gyrus
