# Supplementary material for: Detection, visualization and quantification of protein complexes in human Alzheimer’s disease brains using proximity ligation assay
Source: Sci Rep. 2023 Jul 24;13:11948. doi: 10.1038/s41598-023-38000-4 (PMC10366145; doi:10.1038/s41598-023-38000-4)
Supplement: Supplementary file 1 — Supplementary Information. [file 41598_2023_38000_MOESM1_ESM.pdf]

SUPPLEMENTARY MATERIAL

Proximity Ligation Assay: Detection, Visualization and Quantification of Protein Complexes in Human Alzheimer's Disease Brains

Figure Legend

Table 1. Samples demographics de-identified neuropathological information.

Supplementary Figure 1. Figure shows tau pathology in CA1 of the human hippocampus in "normal aging" non-AD brains compared to aging AD brains with clinically apparent cognitive impairment. A) Non-AD brain sections, *n=4* and B) AD brain sections, *n=4*. The two-channel merged representative images were produced from 10 µm z-stack scanning projections with a step interval of 1 µm. Nuclei were stained with DAPI *(blue)* β-amyloid aggregates and neuritic plaques, neurofibrillary tangles, and other tau aggregates with Thiazine Red *(red)*. The scale bars are indicated.

Supplementary Figure 2. Summary schematic representation of PLA automated data analysis using the General Spot Measurement tool in HCS Studio software associated with the Cell Insight CX7 high-content imaging system. A and B) Quantification of p-tau (Ser202, Thr205)-ubiquitin PLA puncta per field in A) non-AD and B) AD. First, confocal images in RGB format *(left panel)* were split into green (MX-04 signal, *central upper panel*) and red (PLA signal, *central lower panel*) channels and converted to greyscale images using the Image module in the python imaging library Pillow (1, 2, 3). Greyscale images were converted from TIFF to DIB format using the Image Import and Conversion Tool in HCS Studio to generate a new 2 x 1 form factor. Wells A1 and B1 contained the AD images and non-AD images respectively. Samples were analyzed using the General Spot Measurement Tool in HCS Studio. Background removal was performed using the 3D Surface method. For object identification, uniform smoothing was applied to the red channel and a morphology-based detection method was implemented to identify PLA puncta. Fixed thresholding and shape-based segmentation were applied to improve the accuracy of spot detection. PLA puncta were validated by setting conservative intensity and length to width ratio (LWR) limits to eliminate aggregations and background. Yellow object masks indicate valid PLA puncta and orange blots indicate excluded PLA puncta *(right panel)*. C) The AD field contains 1223 PLA puncta, whereas the non-AD field contains 155 PLA puncta.

Table 1. Samples demographics de-identified neuropathological information.

Case ID	Age at death / Sex	Primary clinical categorization	AD neuropathology. Braak & Braak	Other neuropathologies	PMI	Cause of death	Major comorbidities
AD1	68/F	Alzheimer's disease	Severe (A3, B3, C3)	Mild CAA	15h	Dementia	Depression
AD2	70/M	Alzheimer's disease	Severe (A3, B3, C3)	Severe CAA	9.5h	Dementia	None
AD3	71/M	Alzheimer's disease	Severe (A3, B3, C3)	Severe CAA	24h	Hemorrhage/ dementia	Post-hemorrhage seizures, hypertension
AD4	81/M	Alzheimer's disease	Mild (A2, B1, C2)	Severe CAA, 3cm hemorrhage	19h	Dementia/seizure	Atrial fibrillation
AD5	81/F	Alzheimer's disease	Severe (A3, B3, C3)	Severe CAA	36h	Dementia	None
AD6	81/F	Alzheimer's disease	Mild (staging not available)	Severe CAA, remote subdural hemorrhage	48h	Dementia	Atrial fibrillation, seizures, spinal stenosis.
non-AD1	66/F	Neurological control	None	None	33h	Leukemia	COPD, liver transplant recipient
non-AD2	72/M	Neurological control	None	None	12h	Acute respiratory distress syndrome	Renal failure, heart failure.
non-AD3	68/M	Neurological control	None	None	24h	Myocardial infarction	Coronary artery disease
non-AD4	87/M	Non-AD control	None	Stroke (contralateral hemisphere used in study)	6h	Stroke	Arteriolosclerosis
non-AD5	62/M	Neurological control	None	None	11h	Infection	Diabetes, hypertension, renal failure.
non-AD6	68/M	Neurological control	None	None	17h	Infection	Diabetes, hypertension, heart failure.
non-AD7	27/M	Neurological control	None	None	11h	Infection	Heart failure, pulmonary hypertension
non-AD8	45/M	Non-AD	None	Stroke (contralateral hemisphere used in study)	13h	Stroke	Cerebral atherosclerosis, hypertension
non-AD9	87/M	Neurological control	Rare amyloid plaques	None	12h	Myocardial infarction	Coronary artery disease and heart failure

Supplementary Figure 1. *Romero-Fernandez et al., 2023*. Proximity Ligation Assay: Detection, Visualization and Quantification of Protein Complexes in Human Alzheimer's Disease Brains.

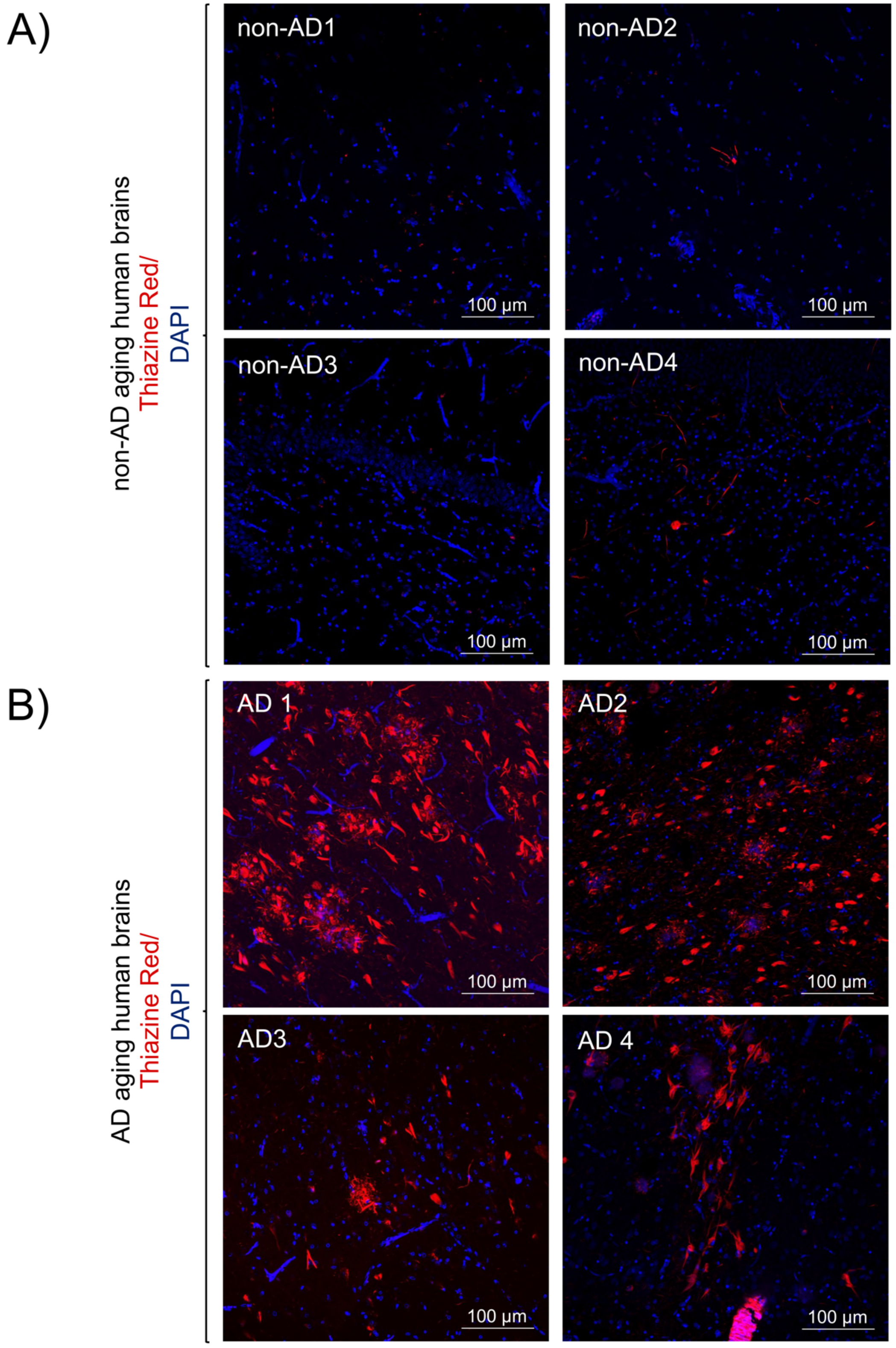

Supplementary Figure 2. *Romero-Fernandez et al., 2023*. Proximity Ligation Assay: Detection, Visualization and Quantification of Protein Complexes in Human Alzheimer's Disease Brains.

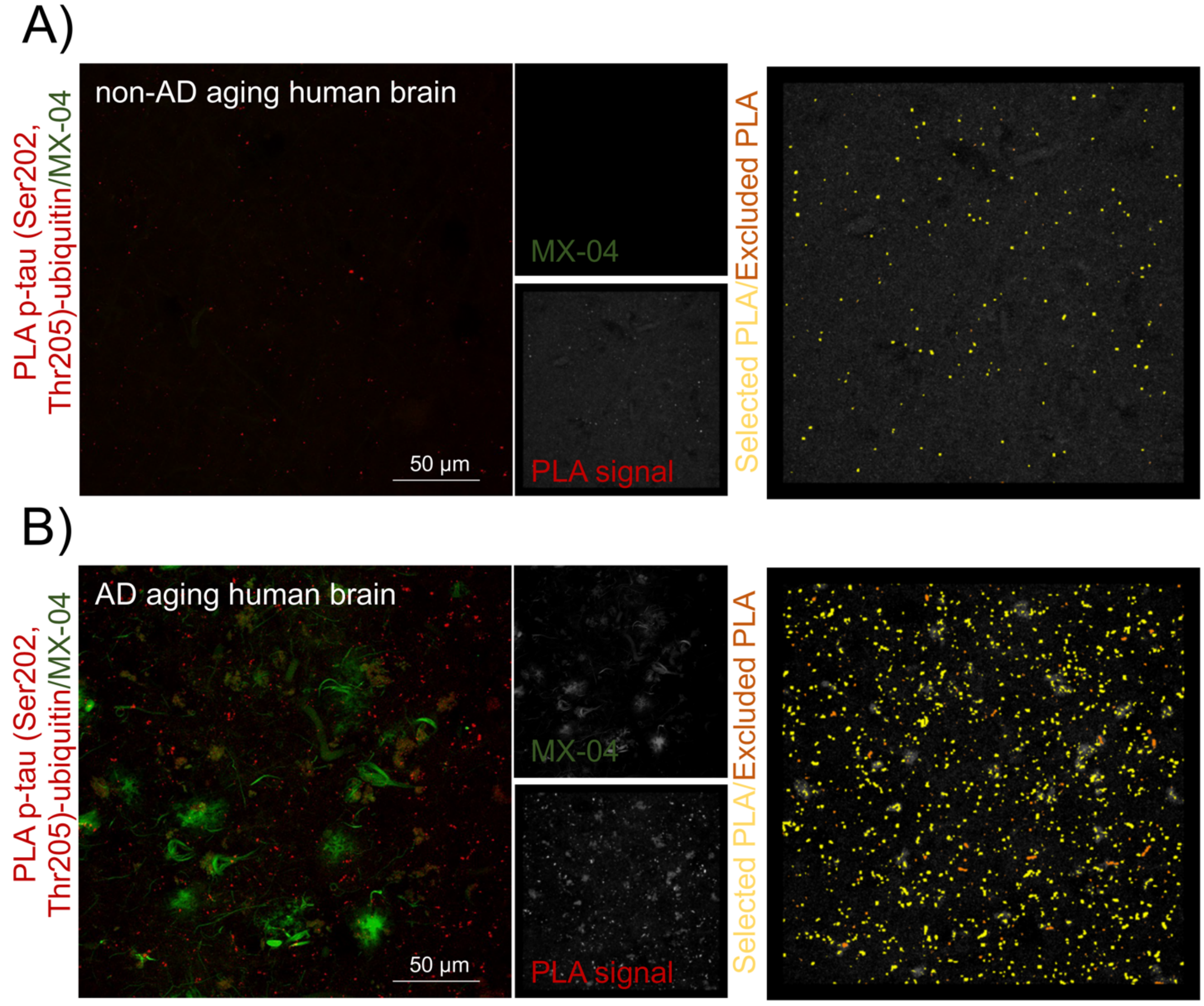

C)

Well	Field	Valid Object Count	Selected Object Count	% Selected Objects	Object Total Area Ch1	Object Avg Area Ch1	Object Total Inten Ch1	Object Avg Inten Ch1	Object Total Inten Per Object Ch1
A1	5	1223	1223	100	2025.016718	1.655778183	177268	14.57196876	144.9452167
B1	28	155	155	100	203.4172158	1.312369134	16026	13.11456628	103.3935484

References:

1. Harris CR, Millman KJ, van der Walt SJ, Gommers R, Virtanen P, Cournapeau D, et al. Array programming with NumPy. Nature. 2020;585(7825):357-62.
2. Van Rossum G, and Drake, F. L. . Python 3 Reference Manual: CreateSpace; 2009.
3. Clark A. Pillow (PIL Fork) Documentation: Readthedocs. Retrieved from https://buildmedia.readthedocs.org/media/pdf/pillow/latest/pillow.pdf; 2015.
